# A conserved influenza A virus nucleoprotein code controls specific viral genome packaging

**DOI:** 10.1038/ncomms12861

**Published:** 2016-09-21

**Authors:** Étori Aguiar Moreira, Anna Weber, Hardin Bolte, Larissa Kolesnikova, Sebastian Giese, Seema Lakdawala, Martin Beer, Gert Zimmer, Adolfo García-Sastre, Martin Schwemmle, Mindaugas Juozapaitis

**Affiliations:** 1 Institute of Virology, University Medical Center Freiburg, 79104 Freiburg, Germany; 2 Spemann Graduate School of Biology and Medicine, University of Freiburg, 79104 Freiburg, Germany; 3 Faculty of Biology, University of Freiburg, 79104 Freiburg, Germany; 4 Institute of Virology, Philipps-Universität Marburg, 35043 Marburg, Germany; 5 Department of Microbiology and Molecular Genetics, University of Pittsburgh School of Medicine, Pittsburgh, Pennsylvania 15217, USA; 6 Institute of Diagnostic Virology, Friedrich-Loeffler-Institut, 17493 Greifswald-Insel Riems, Germany; 7 Institute of Virology and Immunology (IVI), 3147 Mittelhäusern, Switzerland; 8 Department of Microbiology, Icahn School of Medicine at Mount Sinai, New York, New York 10029, USA; 9 Division of Infectious Diseases, Department of Medicine, Icahn School of Medicine at Mount Sinai, New York, New York 10029, USA; 10 Global Health and Emerging Pathogens Institute, Icahn School of Medicine at Mount Sinai, New York, New York 10029, USA

## Abstract

Packaging of the eight genomic RNA segments of influenza A viruses (IAV) into viral particles is coordinated by segment-specific packaging sequences. How the packaging signals regulate the specific incorporation of each RNA segment into virions and whether other viral or host factors are involved in this process is unknown. Here, we show that distinct amino acids of the viral nucleoprotein (NP) are required for packaging of specific RNA segments. This was determined by studying the NP of a bat influenza A-like virus, HL17NL10, in the context of a conventional IAV (SC35M). Replacement of conserved SC35M NP residues by those of HL17NL10 NP resulted in RNA packaging defective IAV. Surprisingly, substitution of these conserved SC35M amino acids with HL17NL10 NP residues led to IAV with altered packaging efficiencies for specific subsets of RNA segments. This suggests that NP harbours an amino acid code that dictates genome packaging into infectious virions.

## Abstract (web summary)

The nucleotide sequence of the eight genomic RNA segments of influenza A virus provides essential packaging signals, but how these sequences are recognized is unknown. Here, Moreira *et al*. identify conserved amino acids in the viral nucleoprotein that regulate packaging of RNA segments.

## Graphical abstract

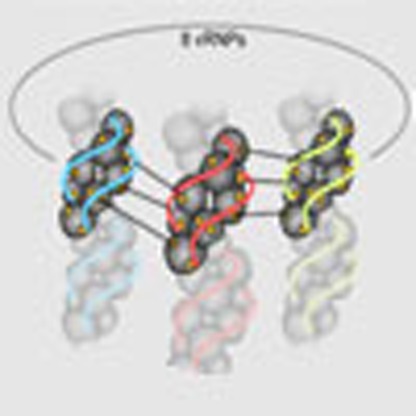

The influenza A virus (IAV) genome is composed of eight negative-sense RNA segments (vRNA), which are encapsidated by multiple copies of the viral nucleoprotein NP^1^. This viral ribonucleoprotein (vRNP) is associated with the polymerase complex consisting of the three subunits PB2, PB1 and PA^1^,^2^. A typical feature of IAV is the exchange of viral genome segments (reassortment) in cells that have been co-infected with at least two different IAV. In avian species, which represent the natural reservoir of IAV, reassortment occurs frequently and affects almost all genome segments^3^,^4^. The exchange of viral genome segments increases the chance for IAV to escape immune pressure from the host or to adapt to new hosts^5^. Indeed, reassortment has often preceded the emergence of pandemic IAV strains in the past^6^. For example, the 2009 pandemic H1N1 virus (pH1N1) originated from a quadruple reassortant virus bearing genome segments from swine, human and avian IAV subtypes^7^,^8^. Likewise, human IAV reassort with co-circulating strains at high frequency, giving rise to seasonal strains that are sometimes more virulent^5^,^9^.

The incorporation of the eight different genome segments into newly formed viral particles seems to be a highly coordinated process. In budding virions, vRNPs form a highly ordered 7+1 arrangement with one of the larger segments usually found in the centre of the bundle^10^,^11^,^12^,^13^. Each of the vRNA segments contains essential packaging sequences encompassing both coding and non-coding regions at the 3′ and 5′ ends. These sequences comprise 50–200 nucleotides (nt), depending on the segment and the virus investigated^14^,^15^. While the sequences in the non-coding regions of the RNA segments are important for the incorporation of the vRNPs into viral particles (also referred to as ‘incorporation signals'), the sequences in the 3′ and 5′ regions of the open reading frames (ORF) seem to be involved in the formation of the 7+1 genome bundle (also referred to as ‘bundling signals')^16^. In addition to these specific packaging sequences, internal short regions have been identified in the viral genome that contribute to genome packaging by interacting with complementary RNA sequences of other segments^17^,^18^. However, it remains to be shown whether vRNA-vRNA interactions play an important role in genome packaging. On the basis of the visualization of vRNAs by fluorescence *in situ* hybridization (FISH) it has been proposed that vRNPs might assemble into bundles at Rab11-positive recycling endosomes *en route* to the plasma membrane^19^,^20^. However, the spatial-temporal coordination of vRNP assembly has not been resolved yet.

Recently, the genomes of two new influenza A-like viruses, provisionally designated HL17NL10 and HL18NL11, have been discovered in bats^21^,^22^. Serological surveys indicated that these two subtypes circulate among different bat species in Central and South America. Bat influenza viruses are distantly related to conventional IAV and share 50–70% identity on the nucleotide level, depending on the segment analyzed^22^. As a consequence of this divergence, only some bat influenza virus-encoded proteins are functionally compatible with conventional IAV proteins. This includes the nucleoprotein (NP) of bat influenza A-like viruses, which fully supports the polymerase activity of several IAV subtypes^23^,^24^. Until now, infectious bat influenza A-like viruses have not been isolated nor have been generated by reverse genetic approaches. However, recombinant bat chimeric viruses containing six gene segments of a bat virus and two segments encoding hemagglutinin (HA) and neuraminidase (NA) of a classical IAV could be rescued *in vitro*^23^,^24^. Of note, packaging of these two segments was only achieved if the authentic 3′ and 5′ genome regions were maintained, suggesting that the packaging sequences of bat influenza A-like viruses and conventional IAV are not compatible. In accordance with this, chimeric bat influenza viruses did not reassort with conventional IAV subtypes H1N1, H3N2 or H7N7, whereas the exchange of segments between bat chimeric viruses of the subtypes HL17NL10 and HL18NL11 was tolerated^23^,^24^. Thus, independent evolution of conventional IAV and bat influenza A-like viruses in their respective hosts might have resulted not only in viral proteins that are mostly functionally incompatible but also in different vRNA packaging sequences.

Here, we show that although bat influenza virus NP fully supported the polymerase activity of conventional IAV, generation of an IAV strain SC35M (H7N7) containing the NP vRNA of HL17NL10 failed. Surprisingly, even when the bat influenza virus NP ORF was flanked with the packaging sequences of SC35M NP vRNA segment no virus could be rescued. Therefore, we generated recombinant viruses with chimeric SC35M and bat NP proteins to tease apart the mechanism behind this incompatibility. Using a mutational approach, we provide evidence that substitution of highly conserved amino acids in the SC35M NP with the corresponding amino acid residues of HL17NL10 NP causes irregular packaging of genome segments. Depending on which amino acids of bat NP are inserted, different sets of viral genome segments were preferentially incorporated into viral particles, suggesting that NPs of conventional IAV harbour a conserved amino acid code that, together with the eight segment-specific RNA packaging sequences, coordinate viral genome packaging.

## Results

### Mutations in the body domain of NP impair genome packaging

**Figure 1:**
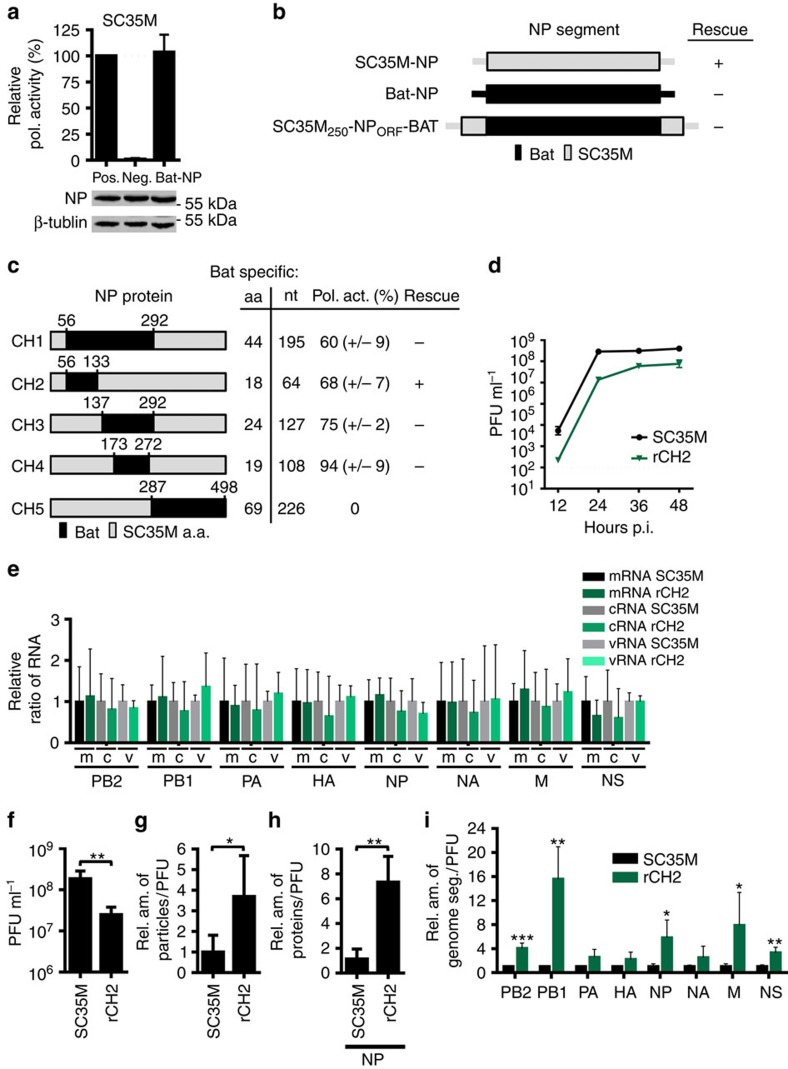
Limited compatibility of SC35M/bat NP chimeras to rescue and package viral genomes of SC35M. (**a**) SC35M polymerase activity in the presence of Bat NP. HEK293T cells were transiently transfected with expression plasmids coding for PB2, PB1, PA of SC35M, the indicated NP proteins, a minigenome encoding the firefly luciferase and a *Renilla* luciferase expression plasmid to normalize for variations in transfection efficiency. In the negative control (Neg.) PB1 was omitted. Western blot analysis was performed to determine the expression levels of NP. (**b**) Cartoon depicting NP segments of A/SC35M (SC35M NP), HL17NL10 (Bat NP) or a NP segment (SC35M_250_-NP_ORF_-Bat) harbouring the non-coding regions of SC35M NP, 5′ and 3′ coding sequences of SC35M NP and the complete ORF of Bat NP. +successful rescue; −no rescue. (**c**) Relative SC35M polymerase activities in the presence of the mutant NP proteins (CH1–CH5). Mean and s.d. of three independent experiments are indicated in parenthesis. SC35M rescue experiments were performed with NP segments encoding the indicated mutant proteins. +successful rescue; −no rescue. (**d**) MDCKII cells were infected at an MOI of 0.001 with wt SC35M or rCH2. At the indicated time points post infection (p.i.), virus titres were determined by plaque assay. (**e**) Relative ratio of the viral transcript level in wt SC35M- or rCH2-infected cells. Steady state levels of viral transcripts (mRNA, cRNA and vRNA) and 5S ribosomal RNA (5S rRNA) were determined by primer extension analysis using total RNA from MDCKII cells infected at an MOI of 5 with wt SC35M or rCH2 for 6 h. Signal intensities were normalized to the signal intensities obtained with 5S rRNA. Normalized values obtained in wt SC35M-infected cells were set to 1 (all non-significant). (**f**) Viral infectivity of SC35M and rCH2 (PFU) using identical HA titre. **P<0.01. (**g**) Relative ratio of the number of viral particles (counted by electron microscopy) divided by the number of infectious particles (determined by plaque assay) between wt SC35M and rCH2. Values obtained for SC35M were set to 1. *P<0.05. (**h**) Ratio of incorporated NP protein in viral particles between SC35M and rCH2. Protein levels were determined by Western blot analysis of virus stocks with equal infectivity (PFU). **P<0.01. (**i**) Relative ratio of genome segments in viral particles preparations of SC35M and rCH2. RNA was prepared from virus stocks with equal PFU and subjected to quantitative RT-PCR. Levels of viral genome transcripts obtained with SC35M were set to 1. *P<0.05; **P<0.01; ***P<0.001. Student's *t* test was used for two-group comparisons. Error bars indicate the mean and s.d. of at least three independent experiments.

Previously, it has been shown that chimeric bat influenza A-like viruses fail to reassort with conventional IAV, including A/SC35M (H7N7), due to an incompatibility of the internal viral proteins as well as differences in the packaging sequences^23^,^24^. Since the bat influenza A-like NP protein (herein referred to as ‘Bat NP') of the HL17NL10 subtype (A/little yellow-shouldered bat/Guatemala/164/2009) supported the polymerase activity of SC35M in a polymerase reconstitution assay (Fig. 1a)^23^, we hypothesized that the generation of recombinant SC35M encoding Bat NP might be possible by employing the packaging sequences of the SC35M NP segment (Fig. 1b). However, in contrast to our expectations such viruses could not be generated (Fig. 1b), even so Bat NP expressed from the pHW2000 rescue plasmid SC35M_250_-NP_ORF_-Bat was expressed and supported SC35M polymerase activity albeit to lower levels than wild-type (wt) SC35M NP (Supplementary Fig. 1). To identify regions of Bat NP that could rescue a recombinant SC35M virus, NP segments and expression plasmids were generated encoding five SC35M/Bat NP chimeras (CH1–CH5) that varied in the amount of Bat NP-specific amino acids and nucleotides (Fig. 1c). Although all SC35M/Bat NP chimeras except CH5 supported polymerase activity of SC35M in polymerase reconstitution assay, only recombinant SC35M encoding the chimera CH2 (herein referred to as ‘rCH2') with 18 Bat NP-specific amino acids located in the so-called body domain of NP (Supplementary Fig. 2) could be successfully generated (Fig. 1c). Infection of MDCKII cells with rCH2 at a multiplicity of infection (MOI) of 0.001 resulted in a 35-fold reduction in viral titres 24 h post infection (h.p.i.) (Fig. 1d), indicating that although the CH2 chimeric NP supported the generation of infectious IAV, the resulting rCH2 virus was attenuated in viral growth. A similar level of attenuation was observed in single cycle infection experiments (MOI of 5) at 6 and 8 h.p.i. with rCH2 (Supplementary Fig. 3). Nevertheless, under the same conditions, comparable mRNA, cRNA and vRNA levels were detected in cells infected with wild-type (wt) SC35M and rCH2 viruses at 6 h.p.i. (Fig. 1e) and in a polymerase reconstitution assay using the SC35M NP segment (Supplementary Fig. 4), indicating that viral polymerase activity and RNA synthesis was not affected by the NP changes in the rCH2 virus, and that this was not the cause for the attenuation of the virus. The small differences of the polymerase activity using the reporter minigenome (Fig. 1c) and the authentic NP segment (Supplementary Fig. 4) are likely to be caused by the artificial nature of the reporter segment. By contrast, at equal hemagglutination titres, supernatants from rCH2 virus-infected cells displayed 10-fold less infectious viruses than wt SC35M (Fig. 1f). Moreover, the ratio of total viral particles to infectious particles, determined by electron microscopy (Supplementary Fig. 5) and plaque assay, was found to be almost fourfold higher in rCH2 than in wt SC35M virus stocks (Fig. 1g). Using equal numbers of infectious viral particles significant higher NP protein levels were detected in rCH2 virus preparations compared with wt SC35M (Fig. 1h). This suggests that relatively more non-infectious viral particles were produced in rCH2-infected cells. To compare the genome equivalents between viral particles released from rCH2 and SC35M-infected cells, we determined the relative ratio of all 8 viral segments by quantitative RT-PCR using an equal number of infectious viral particles as determined by plaque assay. With this approach, the PB2, PB1, NP, M and NS vRNA segments were found to be significantly enriched in the rCH2 preparations compared with the PA, HA and NA segments (Fig. 1i). Together these results suggest that the SC35M/Bat NP chimera rCH2 is characterized by an increased production of non-infectious particles harbouring an irregular set of viral genomes.

**Figure 2:**
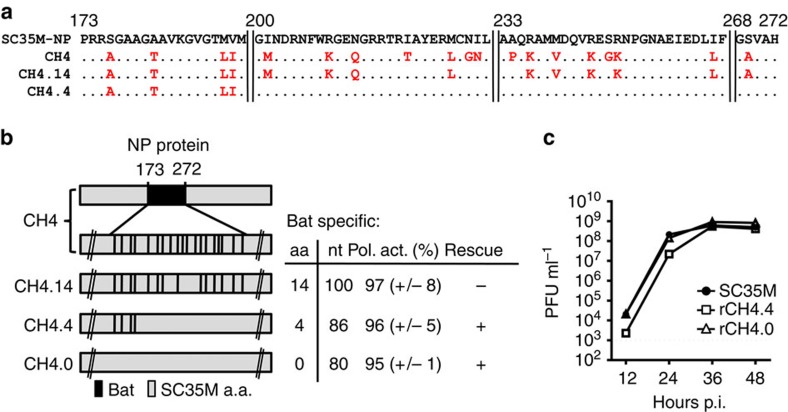
Substitution of 14 Bat NP-specific amino acids in CH4 is required to rescue SC35M. (**a**) Alignment of SC35M NP and CH4 variants. Bat NP-specific amino acids are indicated in red. (**b**) The 19 Bat NP-specific amino acids in CH4 (highlighted in black) were mutated to SC35M-specific amino acids leaving either 14 (CH4.14), 4 (CH4.4) or no Bat NP-specific amino acids (CH4.0) and 100, 86 and 80 Bat NP-specific nucleotides, respectively. Relative polymerase activities of the indicated CH4 variants were determined by polymerase reconstitution assays in HEK293T cells. Mean and s.d. of three independent experiments are indicated in parenthesis. +successful rescue; −no rescue of recombinant viruses. (**c**) MDCKII cells were infected at an MOI of 0.001 with either wt SC35M, rCH4.4 or SC35M-CH4.0. At the indicated time points post infection (p.i.) virus titres were determined by plaque assay. Error bars indicate the mean and s.d. of at least three independent experiments.

To understand why rescue of SC35M with the NP chimeras CH1, CH3 and CH4 failed, we focused on CH4 because it is nested inside both CH1 and CH3, contains only 19 Bat NP-specific amino acids, and harbours the fewest number of Bat NP-specific nucleotides. Moreover, it supported SC35M polymerase activity in polymerase reconstitution assays as efficiently as SC35M NP (Fig. 1c). CH4 was further modified to contain either 14 (CH4.14), 4 (CH4.4) or 0 (CH4.0) Bat NP-specific amino acids (Fig. 2a,b). In CH4.14 the five most divergent residues of the 19 bat NP-specific amino acids with respect to charge and size were replaced with SC35M ones, while only four Bat NP-specific amino acids were left in CH4.4. All NP mutants supported the polymerase activity of SC35M; however, only recombinant SC35M encoding either CH4.4 (rCH4.4) or CH4.0 (rCH4.0) could be successfully generated (Fig. 2b). As expected, rCH4.0 replicated to similar titres as wt SC35M, while rCH4.4 replication was slightly reduced at 12 and 24 h.p.i. (Fig. 2c). These results suggest that the 10 Bat NP-specific amino acids in CH4.14 prevent successful rescue of SC35M.

### Mutations in the head domain of NP affect genome packaging

**Figure 3:**
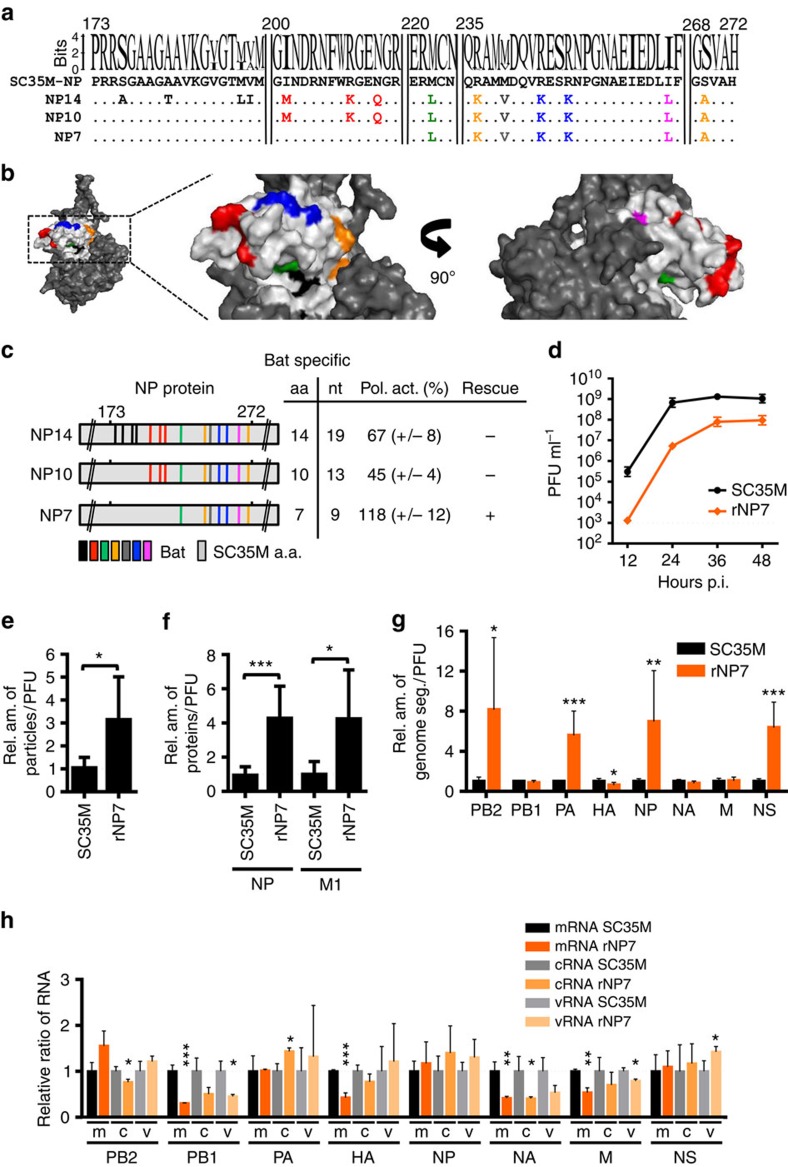
A subset of Bat NP-specific amino acids of CH4 causes severe attenuation and irregular packaging of genome segments. (**a**) Alignment of SC35M NP and NP mutant proteins harbouring 14 (NP14), 10 (NP10) or 7 (NP7) Bat NP-specific amino acids of CH4. Upper panel shows the sequence logo of the consensus sequence of NP of available IAV strains (*n*=27,675 strains). (**b**) Positions of Bat NP-specific amino acids in the modelled crystal structure of SC35M NP. The program PyMOL was used to assign the indicated positions. Bat-specific amino acids are marked in the colour code used in **a**. Note that amino acid 239 is not surface exposed. (**c**) Relative SC35M polymerase activities in the presence of the mutant NP proteins determined by polymerase reconstitution assays. Bat-specific amino acids and nucleotides are indicated. SC35M rescue experiments were performed with NP segments encoding the indicated mutant proteins. +successful rescue; −no rescue. (**d**) MDCKII cells were infected at an MOI of 0.001 with wt SC35M or recombinant virus rNP7. At the indicated time post infection (p.i.), virus titres were determined by plaque assay. (**e**) Relative ratio of the number of viral particles (counted by electron microscopy) divided by the number of infectious particles (determined by plaque assay) between wt SC35M and rNP7. Values obtained for SC35M were set to 1. *P<0.05. (**f**) Ratio of incorporated NP and M1 proteins in viral particles between SC35M and rNP7. Protein levels were determined by Western blot analysis of virus stocks with equal infectivity (PFU). *P<0.05; ***P<0.001. (**g**) Relative ratio of genome segments identified in viral particles preparations of SC35M and rNP7. RNA was prepared from virus stocks with identical infectivity (PFU) and subjected to quantitative RT-PCR. Levels of viral genome transcripts obtained with wt SC35M were set to 1. *P<0.05; **P<0.01; ***P<0.001. (**h**) Relative ratio of the viral transcript level in wt SC35M- or rNP7-infected cells. Steady state levels of viral transcripts (mRNA, cRNA and vRNA) and 5S ribosomal RNA (5S rRNA) were determined by primer extension analysis using total RNA from MDCKII cells infected at an MOI of 5 with wt SC35M or rCH2 for 6 h. Signal intensities were normalized to the signal intensities obtained with 5S RNA. Normalized values obtained in cells infected with wt SC35M were set to 1. *P<0.05; **P<0.01; ***P<0.001. Student's *t* test was used for two-group comparisons. Error bars indicate the mean and s.d. of three independent experiments.

To further analyze the effect of Bat NP-specific amino acids on the production of recombinant SC35M, we first introduced the 14 Bat NP-specific amino acids of CH4.14 into SC35M NP resulting in the SC35M NP14 mutant (Fig. 3a). NP14 was identical on amino acid level with CH4.14 but lacked the large amount of Bat NP-specific nucleotides (Fig. 2b). In addition to NP14, we generated additional SC35M NP mutants with either 10 (NP10) or 7 (NP7) Bat NP-specific amino acids (Fig. 3a). The majority of the inserted Bat NP-specific amino acids are unique and not conserved among conventional IAV strains (Fig. 3a). Most of these residues are found on the surface of the NP head domain (Fig. 3b). NP14, NP10 as well as NP7 supported viral polymerase activity, although to varying degrees (Fig. 3c). However, only recombinant SC35M encoding NP7 (rNP7) could be successfully generated (Fig. 3c). Infection of MDCKII cells with rNP7 at an MOI of 0.001 (Fig. 3d) or 5 (Supplementary Fig. 3) resulted in up to 100-fold lower virus titres compared with wt SC35M. To further determine which of the seven amino acids of NP7 contributed to impaired virus yield, a series of NP7 variants lacking either individual or clusters of Bat NP-specific amino acids was generated (Supplementary Fig. 6a). All of these mutants supported polymerase activity and in all cases SC35M viruses containing these NP mutants could be successfully generated (Supplementary Fig. 6b). SC35M virus encoding a NP7 variant with only six Bat NP-specific mutations, designated rNP7(2-7), was still attenuated in MDCKII cells and replicated to 50-fold lower virus titres than wt SC35M at 24 h.p.i. (Supplementary Fig. 6c). In contrast, replication of the other rNP7 variants was not significantly attenuated (Supplementary Fig. 6c). These findings suggest that attenuation of rNP7 is caused by several Bat NP-specific amino acids acting in a concerted manner.

Similar to rCH2, the impaired viral growth of rNP7 might be linked to a deficit in genome packaging. Indeed, the ratio of total viral particles to infectious particles, determined by electron microscopy (Supplementary Fig. 5) and plaque assay, was found to be threefold higher in rNP7 than in wt SC35M virus stocks (Fig. 3e). Using equal numbers of infectious viral particles significant higher NP and M1 protein levels were detected in rNP7 virus preparations compared with wt SC35M (Fig. 3f). Interestingly, we also found significantly higher levels of four viral genome segments, PB2, PA, NP and NS, in rNP7 particle preparations (Fig. 3g). These observations suggest that an irregular number of genome segments was incorporated into the viral particles following infection of cells with rNP7. Compared with wt SC35M, infection of MDCKII cells with rNP7 (MOI of 5) resulted in slightly lower levels of mRNA, cRNA and vRNA (Fig. 3h) of the PB1, M and NA segments at 6 h.p.i. These segments were underrepresented in the viral particles (Fig. 3g). At high MOI, the differences in the segment-specific composition of non-infectious rNP7 and rCH2 viral particles might account for the lower expression levels of mRNA, cRNA and vRNA of some genes in rNP7-infected cells. In addition, no alterations in the subcellular localization of M1, NP or HA were observed between cells infected with wt SC35M and rNP7 during the course of infection (Supplementary Fig. 7).

**Figure 4:**
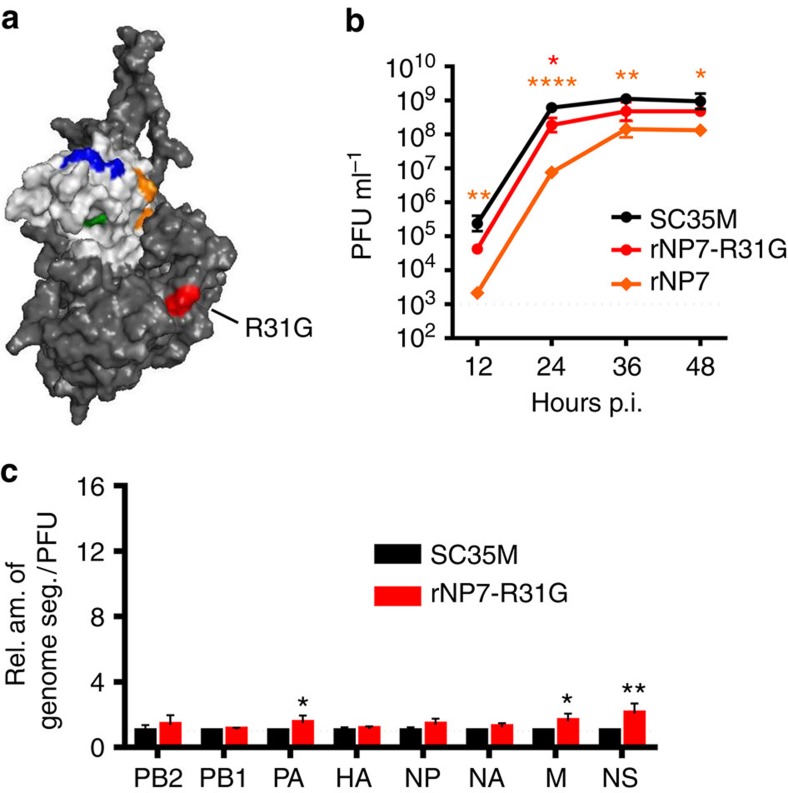
The compensatory amino acid substitution R31G in NP7 improves viral growth and genome packaging. (**a**) Positions of Bat NP-specific amino acids in the head domain and R31G body domain of NP7 using the modelled crystal structure of SC35M NP. The program PyMOL was used to assign the indicated positions. Bat-specific amino acids are marked in the colour code used in Fig. 3b. (**b**) MDCKII cells were infected at an MOI of 0.001 with wt SC35M, rNP7 or rNP7-R31G. At the indicated time post infection (p.i.), viral titres were determined by plaque assay. *P<0.05; **P<0.01; ****P<0.0001. (**c**) Relative ratio of genome segments identified in viral particles preparations of SC35M and rNP7-R31G. RNA was prepared from virus stocks with identical infectivity (PFU) and subjected to quantitative RT-PCR. Levels of viral genome transcripts obtained with wt SC35M were set to 1. *P<0.05; **P<0.01. Student's *t* test was used for two-group comparisons. Error bars indicate the mean and s.d. of at least three independent experiments.

Serial passage of rNP7 in MDCKII cells resulted in a virus strain that replicated almost as efficiently as wt SC35M. Sequencing of the NP ORF revealed that the passaged rNP7 virus contained a single point mutation in the body domain of NP at position 31 (R31G) (Fig. 4a), suggesting that this mutation improved viral growth. In agreement with this hypothesis, a recombinant rNP7 harbouring this additional mutation (rNP7-R31G) replicated in MDCKII cells to markedly higher viral titres than rNP7 (Fig. 4b). We speculated that this mutation might also restore the packaging of the vRNA segments. Indeed, using equal numbers of infectious SC35M and rNP7-R31G viruses, a similar proportion of viral genome segments was detected in rNP7-R31G and wt S35M (Fig. 4c).

In summary, these results indicate that substitution of conserved amino acids in the head domain of SC35M NP with Bat NP-specific amino acids resulted in attenuation of SC35M to variable degrees. As demonstrated by the virus mutant rNP7, attenuation was correlated with an increased production of non-infectious particles that incorporated a restricted set of genome segments. This attenuation could be partially overcome by the compensatory amino acid mutation R31G in NP. Most interestingly, the introduction of Bat NP-specific mutations into the head or body domain of SC35M NP resulted in the accumulation of viral particles with distinct sets of viral RNA segments (compare Fig. 1i and Fig. 3g).

### NP7 and CH2 inefficiently package all eight viral genomes

The observation that cells infected with either rCH2 or rNP7 released large numbers of viral particles with an incomplete set of viral genomes suggested that both NP proteins CH2 and NP7 fail to support coordinated packaging of all eight segments. To demonstrate this, we made use of a recently published virus-like particle (VLP)-based RNA segment packaging assay^16^. In this assay, an NP segment based reporter minigenome encoding green fluorescent protein (GFP) is efficiently packaged in the presence of the seven remaining viral genome segments only if the reporter is flanked with both the non-coding regions also known as incorporation signals (IS) and the additional packaging sequences located in the terminal 3′ and 5′ regions of the NP ORF, which have been designated genome bundling sequences (BS)^16^. The IS are believed to be required for the incorporation of the genome segments into viral particles, while the BS have been proposed to be responsible for correct bundling of all eight viral genome segments^16^.

**Figure 5:**
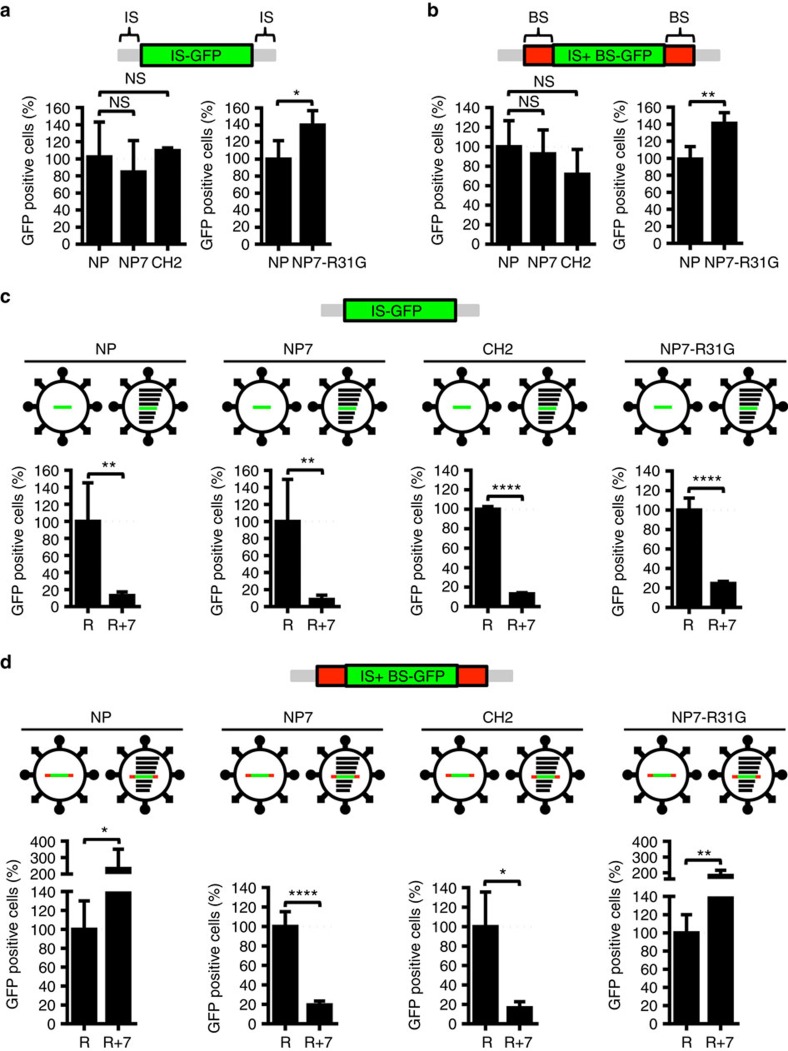
Bat NP-specific amino acids in SC35M NP cause impaired packaging. (**a**,**b**) Packaging efficiency of a single minigenome supported by SC35M NP (NP), CH2, NP7 or NP7-R31G in a VLP-based packaging assay. Reconstitutions of SC35M VLPs were carried out in the presence a GFP-encoding reporter segment flanked by IS of the NP segment (IS−GFP) or in addition with the bundling signals (BS) in the 5′ and 3′ NP ORF (IS+BS−GFP). After infection of MDCKII cells and subsequent superinfection with wt SC35M, GFP-positive cells were quantified by FACS. NS, not significant; *P<0.05; **P<0.01. (**c**,**d**) Packaging efficiency mediated by SC35M NP (NP), NP7, CH2 or NP7-R31G in the presence of the reporter segment IS+GFP (R) (**c**) or IS+BS−GFP (R) (**d**) and, if indicated, in the presence of the remaining seven wild-type genome segments (+7). NS, not significant; *P<0.05; **P<0.01; ****P<0.0001. Student's *t* test was used for two-group comparisons. Error bars indicate the mean and s.d. of at least three independent experiments.

We constructed a GFP reporter minigenome containing either the IS of the SC35M NP segment (IS−GFP) or both the IS and BS elements (IS+BS−GFP) and compared the efficacy of CH2 and NP7 NP proteins to package these reporter minigenomes into viral particles in the absence of the other seven segments. For this purpose, SC35M VLPs were generated in human 293 T cells in the presence of the reporter minigenome (IS−GFP or IS+BS−GFP) and NP protein, either wt SC35M NP or mutant CH2 or NP7. The incorporation of the GFP reporter minigenomes into VLPs was subsequently quantified by co-infection of MDCKII cells with VLPs and wt SC35M virus. No differences were observed in the amount of VLPs released from 293 T cells as evidenced by the number of GFP-positive MDCKII cells (Fig. 5a,b). This finding indicates that the incorporation of a single genome segment into viral particles is not affected, irrespective of the presence of the bundling sequence.

We next tested the efficacy with which the reporter minigenomes were incorporated into VLPs in the presence of the seven remaining genome segments. As expected, infection of MDCKII cells with VLPs reconstituted with wt SC35M NP along with a reporter segment lacking the BS (IS−GFP) and all seven wt SC35M genome segments resulted in significantly lower numbers of GFP-positive cells than infection with VLPs reconstituted without the seven genome segments (Fig. 5c). Similar results were obtained with both CH2 and NP7 proteins (Fig. 5c). Infection of MDCKII cells with VLPs reconstituted with SC35M NP, seven wt SC35M genome segments, and the IS+BS−GFP reporter minigenome resulted in significantly higher numbers of GFP-positive cells compared with infections with VLPs reconstituted with the reporter minigenome only (Fig. 5d). In contrast, CH2 and NP7 NP proteins were unable to efficiently reconstitute VLPs in the presence of all seven wt SC35M genome segments (Fig. 5d). As expected, the R31G mutation in NP7 (NP7−R31G) was a reversion that almost restored the packaging efficiency to wt SC35M NP levels (Fig. 5d). Using this VLP-based packaging assay, we also found that wt Bat NP in combination with the additional seven wt SC35M genome segments did not mediate efficient packaging of an IS+BS−GFP reporter minigenome (Supplementary Fig. 8). Taken together, these results strongly suggest that the attenuation of SC35M virus following the introduction of Bat NP-specific amino acids into SC35M NP is due to a defect in packaging of a full complement of eight viral RNA segments and that the incompatibility between human IAV and bat influenza A-like viruses might be a consequence of this packaging defect.

## Discussion

In this study, we altered highly conserved amino acid residues in the NP protein of the conventional SC35M IAV with residues only present in the NP protein of a newly discovered bat influenza A-like virus (Bat NP)(Fig. 3a,b; Supplementary Fig. 2). We provide for the first time evidence for the important role of NP-specific amino acid residues in mediating efficient packaging of all eight genome RNA segments into viral particles. We refer to this hitherto unrecognized set of amino acids as the ‘NP packaging code'. These amino acid residues seem to be important for the coordinated packaging of multiple segments, but not for incorporation of a single segment into virus particles. Moreover, we have identified amino acids in NP that have a distinct function in genome packaging, but do not impair viral polymerase activity and RNA synthesis.

Although the full breadth of this putative ‘NP packaging code' needs to be determined, our data suggest that the amino acids comprising this code are at least present in both the head and the body domain of NP. Whether other highly conserved amino acids in NP are part of this NP packaging code remains to be determined. Analysis of conserved amino acids at the C-terminus of IAV NP using SC35M/Bat NP chimeras was not possible, since such fusion proteins turned out to be inactive in our polymerase reconstitution assays. Previous studies exploring the importance of highly conserved NP amino acid residues between influenza A, B and C viruses identified single amino acids in the head domain that abrogated viral growth, but still allowed packaging of at least one but not eight genome segments using a VLP-based packaging assay^25^. Although packaging in the context of a viral infection could not be studied, these single amino acids might also constitute essential key residues of the ‘NP packaging code'.

**Figure 6:**
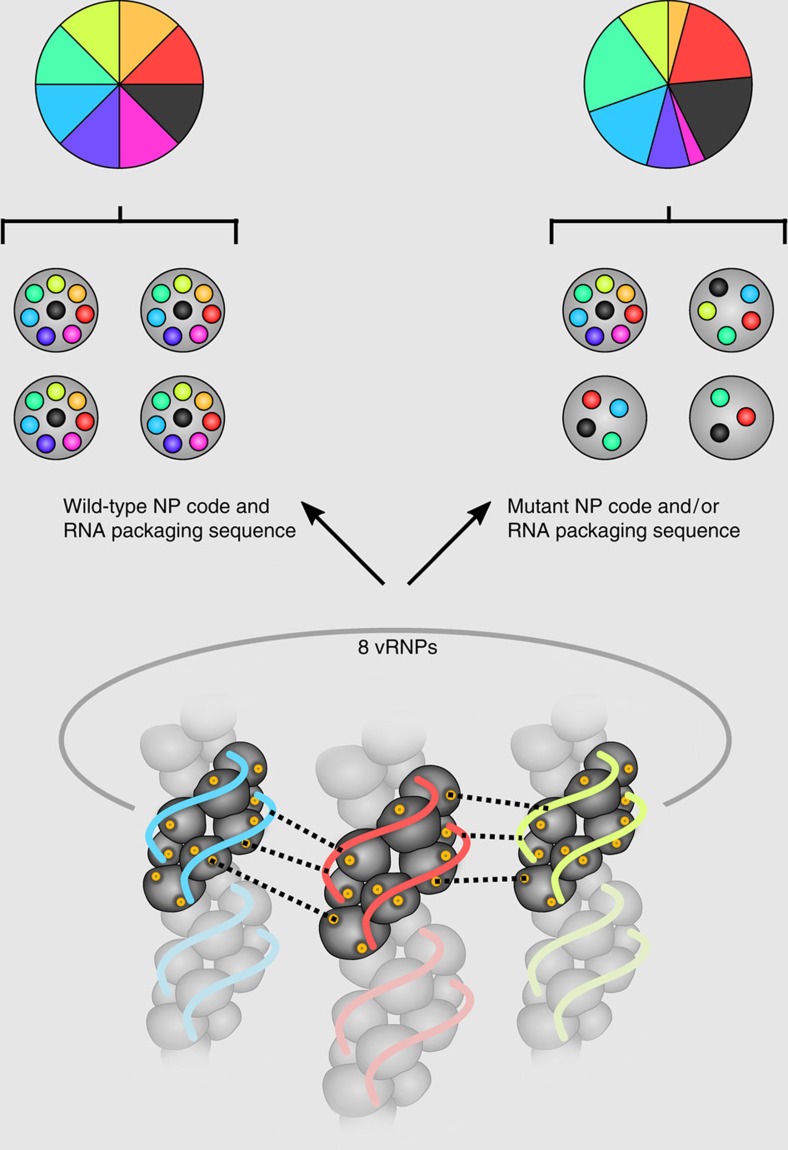
Genome packaging sequence or NP code mutations disrupt coordinated packaging of eight influenza genome segments into viral particles. Wt NP code and vRNA packaging sequences ensure coordinated incorporation of the eight different genome segments into influenza A virus particles, resulting in an equal ratio of the individual viral genome segments (left panel). As indicated by dashed lines, packaging could be coordinated by various interactions between segment-specific vRNPs, including interactions between vRNA packaging sequences, between vRNA packaging sequences in one vRNP and amino acids of the NP code (indicated in yellow) in a second vRNP, and direct interactions of the NP code amino acids. Mutations in the NP code and/or vRNA packaging sequence result in the loss of coordinated packaging of the eight different genome segments into viral particles and a disproportional ratio of the viral segments (right panel). Loss of coordinated packaging might be caused by impaired interactions between vRNPs mediated by amino acids of the NP code and/or nucleotides of the RNA packaging sequences.

Surprisingly, mutation of conserved amino acid residues in the NP body domain resulted in the incorporation of a different subset of viral genome segments than those seen with alteration of the NP head domain. Similarly, irregular genome packaging has been observed after mutating packaging signal sequences of IAV genomes^18^,^26^,^27^,^28^,^29^,^30^,^31^,^32^. Depending on the nucleotide mutations introduced into individual packaging sequences, coordinated packaging was lost and different sets of viral genomes were incorporated into viral particles. Thus disruption of either the RNA packaging sequences or amino acids of the NP packaging code can block coordinated packaging of the eight genome segments and, as a consequence, may cause impaired release of infectious viral particles (Fig. 6). Although formal proof is still missing, this might suggest that the ‘NP packaging code' is complex and has to match to individual genome packaging sequences in order to coordinate the incorporation of a full complement of eight genome segments into budding viral particles. Since RNA loop regions are believed to interact with each other thereby orchestrating the coordinated packaging of the different genome segments^14^, it is tempting to speculate that the ‘NP packaging code' provides the required vRNP conformations that facilitate RNA loop interactions between different vRNPs.

Conventional IAV share both highly compatible packaging sequences and functionally exchangeable NP proteins, thereby enabling genome reassortment among all known IAV subtypes tested so far^5^,^6^,^15^,^33^,^34^,^35^,^36^,^37^. Similarly, the known bat influenza A-like virus subtypes also share compatible packaging sequences, compatible NP proteins, and the ability to reassort among them^23^,^24^. However, bat influenza A-like viruses, unlike conventional IAV, circulate in bat species and developed RNA packaging sequences and an NP packaging code, which are incompatible with those of conventional IAV^23^,^24^. This suggests that co-evolution of the specific RNA packaging signals and the ‘NP packaging code' resulted in optimal interactions between the eight viral RNA segment bundles during the process of genome packaging. Thus, independent viral evolution in different natural hosts^21^ was probably the driving force that shaped the genome packaging sequences and the ‘NP packaging code' for different Orthomyxoviruses, including influenza B viruses.

Our study suggests that besides the known RNA packaging sequences, conserved residues on the NP protein are essential for coordinated incorporation of the eight different IAV genome segments into viral particles. This finding might not only pave the way to understand the functional interactions of the packaging sequences and NP, resulting in infectious IAV with a full complement of viral RNAs, but also highlights novel attractive targets in the NP for the development of new antivirals that inhibit full viral genome assembly. Finally, the discovery of an amino acid code in an RNA binding protein, such as the one described here for the influenza virus NP that coordinates intricate RNA–RNA interactions leading to specific RNA complexes might be a more general principle applicable to the assembly of other functional multi-RNA complexes.

## Methods

### Plasmids

pHW2000 rescue vectors to generate recombinant SC35M, bat HL17NL10 NP genome and pCAGGS plasmids coding for SC35M proteins or Bat NP have been previously described^23^,^38^. For generation of the SC35M_250_-NP_ORF_-Bat pHW2000 rescue vector, the 3′ and 5′ non-coding regions of the Bat NP genome segment were replaced with nucleotides 1–141 and 1,444–1,565 of the SC35M NP segment. In addition, ATG codons in the 5′ coding sequence of the newly inserted SC35M NP ORF were mutated to ACGs to prevent initiation of translation at these sites. The NP chimeras encoding pHW2000 rescue vectors (Supplementary Table 1) were generated by assembly PCR using various primers (Supplementary Table 2). The NP chimeras were re-cloned into pCAGGS plasmids via internal ORF cloning sites. All sequences of newly generated NP chimeras were deposited in the GenBank database (Supplementary Table 1) In NP chimera CH1, CH2, CH3, CH4 and CH5 the parts of SC35M NP ORF of different lengths were replaced with sequences encoding corresponding parts of the Bat NP (Supplementary Table 3). To generate SC35M NP genome segment based reporters, the GFP protein coding sequence was fused by assembly PCR either with 5′ and 3′ non-coding regions (NCRs) of SC35M NP genome segment (denoted hereafter as IS) or with NCRs plus 60 and 120 nt comprising 5′ and 3′ ends of the NP ORF (denoted hereafter as IS+BS), respectively. The newly generated reporters were cloned into a pHW400 vector allowing polI-driven expression of the reporter minigenome. pHW400 was generated by removing the polII promoter and terminator from the pHW2000 rescue vector. The same pHW400 vector was used to generate SC35M PB2, PB1, PA, HA, NA, M and NS genome segments.

### Cell lines

Canine MDCKII^39^ and HEK293T cells^40^ were mycoplasma free and maintained in Dulbecco's modified Eagle's medium (DMEM) supplemented with 10% fetal calf serum (FCS), 2 mM L-glutamine, 100 U penicillin per ml and 100 mg streptomycin per ml.

### Infection of cell cultures

MDCKII cells were washed with phosphate-buffered saline (PBS) and infected with viruses diluted in PBS containing 0.2% bovine serum albumin (BSA). The infection solution was replaced 1 h.p.i. with DMEM containing 0.2% (v/w) BSA, 2 mM L-glutamine, 100 U penicillin per ml and 100 μg streptomycin per ml and further incubated at 37 °C. For single cycle infection assays, cells were first incubated with viruses diluted in PBS containing 0.2% BSA at 4 °C. After 1 h, cells were incubated for 10 min at 37 °C, washed with PBS, incubated in PBS (pH=2) for 45 s, washed again with PBS and further incubated at 37 °C in DMEM containing 0.2% (v/w) BSA, 2 mM L-glutamine, 100 U penicillin per ml and 100 μg streptomycin per ml for the indicated time points.

### Formation of VLPs

VLPs were generated essentially as described^41^. Briefly, HEK293T cells seeded in six-well plates were transfected with 1 μg of pCAGGS expression plasmid coding for PB2, PB1, HA, NA, NP and NEP, 0.1 μg of pCAGGS expression plasmid coding for PA and M2, and 2 μg of pCAGGS expression plasmid coding for M1, plus 1 μg of a GFP-encoding minigenome harbouring either IS or IS+BS of SC35M NP genome segment, using the Lipofectamin2000 transfection reagent (PAA Laboratories) in 2 ml of Opti-MEM (Invitrogen) according to the manufacturer's protocol. Culture medium was replaced by DMEM containing 0.2% BSA 8 h post transfection. After 48 h, 1 ml of cell supernatant was transferred to MDCKII cells in six-well plates, infected with A/SC35M (MOI of 5) and incubated for further 10 h. GFP signals were monitored by live imaging. At 10 h post infection, the cells were rinsed with PBS and trypsinized to prepare a single cell suspension containing 1% BSA. GFP expression was analyzed in an FL1 detector of FACSCalibur (Becton Dickinson). To produce VLPs in the presence of the remaining seven wt genome segments, HEK 293 T cells were transfected with 0.2 μg of GFP-encoding minigenome plus 0.2 μg of each of the seven polI (pHW400) plasmids, for vRNA generation, together with the described set of plasmids required for viral protein production.

### Polymerase reconstitution assay

The polymerase reconstitution was essentially carried out as described^38^,^42^. pCAGGS plasmids encoding PB2, PB1, PA (each 50 ng) and NP (200 ng) were co-transfected with the firefly luciferase-encoding viral minigenome construct pPolI-FFLuc-RT (200 ng) and plasmid (pRL-SV40; 50 ng) coding for renilla luciferase in HEK 293 T. Firefly and renilla luciferase activities were measured using the dual luciferase reporter assay 24 h post transfection.

### Virus rescue

The recombinant SC35M/bat chimeras were generated by the eight plasmid reverse-genetics system^38^. The viral cDNA is inserted between the RNA polymerase I (polI) promoter and terminator sequences and the entire polI transcription unit is flanked by an RNA polymerase II (polII) promoter and a polyadenylation site. All viruses were plaque purified and viral titres were determined by plaque assay on MDCKII cells. The introduced changes in the NP segment were confirmed by sequencing.

### Primer extension analysis

Primer extension was essentially carried out as described^42^ with few modifications. For determination of viral transcript levels of all eight influenza segments, confluent MDCKII cells were infected for 1 h in 6-well plates at 4 °C. The cells were then incubated for further 10 minutes at 37 °C before being washed with PBS and treated with PBS (pH=2) for 45 s, washed with PBS and further incubated at 37 °C in DMEM containing 0.2% (v/w) BSA, 2 mM L-glutamine, 100U penicillin per ml and 100 μg streptomycin per ml. At 6 h p.i., cells were collected in 1 ml of TRIzol reagent (Invitrogen) and RNA was purified with Direct-zol™ RNA MiniPrep Kit (Zymo Research) according to the manufacturer's protocol. Primer extension analysis was performed as described before^42^ using specific primers for the influenza virus segments and cellular ribosomal RNA (5S RNA: 5′- TCCCAGGCGGTCTCCCATCC -3′). The primers used for primer extension analysis are specified in Supplementary Table 4. The specific RNA bands were visualized after exposing the gel overnight to a phophoimage plate and quantified using ImageJ^43^. Uncropped scan of the primer extension data can be found in Supplementary Fig. 9.

### Western blot analysis

Proteins from cell lysates were separated by 10% acrylamide SDS–PAGE and subjected to western blot analysis using polyclonal rabbit anti-NP antibody (catalogue number: PA5-32242; Thermo Fisher; diluted 1:2,500), monoclonal mouse anti-β-tubulin antibody (catalogue number: T4026; Sigma-Aldrich; diluted 1:1,000) and the corresponding secondary antibodies IRDye 680RD goat anti-rabbit IgG (H+L) (LI-COR Biosciences; diluted 1:5,000) or IRDye 800CW goat (polyclonal) anti-mouse (LI-COR Biosciences; diluted 1:5,000). Signals were visualized by Odyssey Imaging System (LI-COR Biosciences). Proteins of purified viral particles were separated as described above and detected by western blot analysis using polyclonal rabbit anti-NP, polyclonal mouse anti-M1 (provided by Dr. Jovan Pavlovic; diluted 1:100) and corresponding peroxidase-conjugated goat anti-mouse IgG (H+L) (Jackson ImmunoResearch; diluted 1:5,000) or peroxidase-conjugated goat anti-rabbit IgG (H+L) (Jackson ImmunoResearch; diluted 1:5,000) antibodies. Signals were detected with an Odyssey Imaging System (LI-COR Biosciences). Uncropped western blots can be found in Supplementary Fig. 9.

### Immunofluorescence

After the indicated time post infection, the cells on glass slides were fixed with paraformaldehyde 4% for 10 min, washed with PBS, permeabilized with 0.5% Triton X-100 in PBS for 5 min (for H7 extracellular staining cells were not permeabilized) and incubated with antibodies against NP (catalogue number: PA5-32242; Thermo Fisher; 1:2000), H7 (ref. 44) (1:100), or M1 (provided by Dr. Jovan Pavlovic; 1:10) for 1 h at room temperature. After washing with PBS and incubation with corresponding secondary antibody Cy3 goat anti-rabbit IgG (H+L) (Jackson ImmunoResearch; diluted 1:200), Alexa Fluor 488 donkey anti-chicken IgY (IgG) (H+L) (Jackson ImmunoResearch; diluted 1:500) or Cy3 goat anti-mouse IgG (H+L) (Jackson ImmunoResearch; diluted 1:200) for 30 min at room temperature, nuclei were stained for 5 min with DAPI (diluted 1:10,000 in PBS). The glass slides were examined on an Axioplan 2 Imaging System (Carl Zeiss, Oberkochen, Germany) equipped with an ApoTome.

### qPCR analysis of packaged vRNAs

The qPCR analysis of packaged vRNAs has been described previously^45^. Briefly, to analyze packaged vRNAs, virus stocks were prepared and adjusted to equal plaque forming units (PFU) titres. Genomic vRNAs were extracted by Direct-zol RNA MiniPrep (Zymo Research) and eluted in 50 μl of water. Overall, 5 μl of RNA were reverse transcribed using RevertAid H Minus Reverse Transcriptase (Thermo Scientific) using two primers (5′- AGCAAAAGCAGG -3′ and 5′- AGCGAAAGCAGG -3′). The RT product was then diluted (1:5) and used as template for quantitative PCR with SensiFAST SYBR Hi-ROX Kit (Bioline) and the 7300 Real Time PCR System (Applied Biosystems). The SC35M segment-specific PCR primers used are indicated on Supplementary Table 5. Relative concentrations of vRNAs were determined on the basis of analysis of cycle threshold values of standard curves designed for each viral segment consisting of serial 1:10 dilutions of a wt SC35M cDNA. The incorporation of vRNA for each segment of SC35M was then compared with the corresponding segment of the chimeric viruses. Results are presented as the average incorporations of vRNA±s.d., resulting from at least three independent virus stocks.

### Multiple alignment

A total of 27,675 sequences of NP protein of influenza A isolates were downloaded from National Center for Biotechnology Information database and aligned in MEGA6 (ref. 46). The consensus was illustrated as sequence logo using Geneious software suite v. 6.1.8.

### Hemmaglutination assay

A volume of 50 μl of a working solution of 0.25% red blood cells from chicken was added to the serially 1:2 diluted virus of interest in a round-bottomed 96-well dish and kept at room temperature for 30–60 min to develop. The HA titre of the corresponding virus was determined as the number of the highest dilution factor that produced a uniform reddish colour across the well.

### Particle counting by transmission electron microscopy

Viral supernatant from MDCKII-infected cells was fixed with 4% paraformaldehyde, and after virus inactivation by fixation was combined with an equal volume of polysterene beads of a known concentration (7.08 × 10^11^ per ml, 137 nm in diameter, Plano, Germany). The virus-bead mixtures were deposited onto Formvar-coated 400-mesh grids pretreated with 1% alcian blue and allowed to adhere for 10 min. Then, grids were negatively stained with 2% phosphotungstic acid. The viral particles and beads were counted in 10 different randomly chosen squares using a JEM 1400 transmission electron microscope (TEM). The total number of virus particles per millilitre was determined according equation: (mean value of virus particles per square) × (concentration of latex beads)/(mean number of latex beads per square). The number of viral particles present per millilitre was divided by the number of PFU per millilitre to yield the particle/PFU ratio.

### Molecular modelling

The program I-TASSER (zhanglab.ccmb.med.umich.edu/I-TASSER) was used to generate full-length models of CH2 and NP7. The program PyMOL ( www.pymol.org) was used to assign the indicated positions in the generated structural models.

### Structural analysis by transmission electron microscopy

For analysis of virus stocks, confluent MDCKII cells were infected with SC35M, rCH2, or rNP7 in 6-well plates at an MOI of 0.001. At 24 h.p.i. 10 ml of culture supernatant of infected MDCK II cells were centrifuged at 780*g* for 5 min to remove cell debris and then ultracentrifuged at 90,000*g* for 1.5 h at 4 °C. The pelleted viral particles were suspended in 200 μl of PBS and fixed with 4% paraformaldehyde. After the fixation viral samples were contrasted with uranyl acetate and lead citrate and analyzed with a JEM 1400 transmission electron microscope at 120 kV. The representative images for each preparation were acquired using a TVIPS TemCam F416 camera. For ultrathin section analysis of infected cells, confluent MDCKII cells were infected with wt SC35M, rCH2 or rNP7 for 1 h in 6-well plates at 4 °C at an MOI of 10. At 24 h.p.i. cells were fixed directly in wells with 4% PFA and 0.1% glutaraldehyde in a 100 mM PHEM buffer (60 mM piperazine-N,N′-bis(2-ethanesulfonic acid) (PIPES), 25 mM HEPES, 2 mM MgCl_2_, 10 mM EGTA (pH 6.9)) for 60 min at room temperature. The method of in situ pre-fixation allowed preserving virus particles localized on the cell surface. Then, cells were scraped, pelleted and incubated overnight with 4% PFA in the 100 mM PHEM buffer at 4 °C. After washing with a 100 mM HEPES buffer, cells were post-fixed for 60 min with 1% osmium tetroxide in the 100 mM HEPES buffer (pH 7.4), dehydrated in ultra-pure grade ethanol, embedded in a mixture of Epon and Araldite, and polymerized at 60 °C for 24 h. Ultrathin sections (60–90 nm) of the cells were cut with a Leica EM UC6 microtome. The sections were contrasted with uranyl acetate and lead citrate and analyzed with TEM as described above for the analysis of virus stocks.

### Statistics

Student's *t* test was used for two-group comparisons. The *P value <0.05, **P value <0.01, ***P value <0.001 and ****P value <0.0001 were considered significant. Error bars indicate the mean and s.d. of at least three independent experiments.

### Data availability

The authors declare that all data supporting the findings of this study are available within the article and its Supplementary Information files, or from the authors upon request. The DNA sequences of the NP chimeras used in this study were deposited in the GenBank database and the accession codes are indicated in Supplementary Table 1.

## Additional information

**How to cite this article:** Moreira, E. A. *et al*. A conserved influenza A virus nucleoprotein code controls specific viral genome packaging. *Nat. Commun.* 7:12861 doi: 10.1038/ncomms12861 (2016).

## Supplementary Material

Supplementary Information Supplementary Figures 1-9 and Supplementary Tables 1-5
