# Supplementary material for: A conserved influenza A virus nucleoprotein code controls specific viral genome packaging
Source: Nat Commun. 2016 Sep 21;7:12861. doi: 10.1038/ncomms12861 (PMC5035998; doi:10.1038/ncomms12861)
Supplement: Supplementary Information — Supplementary Figures 1-9 and Supplementary Tables 1-5 [file ncomms12861-s1.pdf]

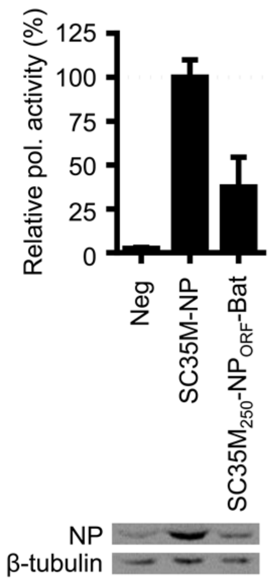

Supplementary Figure 1. SC35M polymerase activity in the presence of Bat or SC35M NP encoded from the pHW2000 rescue plasmid.

HEK293T cells were transiently transfected with expression plasmids coding for PB2, PB1, PA of SC35M, the indicated NP proteins encoded by pHW2000 plasmids harboring the packaging sequences of SC35M (see also Fig. 1b), a minigenome encoding the firefly luciferase and a *Renilla* luciferase expression plasmid to normalize for variations in transfection efficiency. In the negative control (Neg) PB1 was omitted. Error bars indicate the mean and SD of at least 3 independent experiments. Western blot analysis was performed to determine the expression levels of NP.

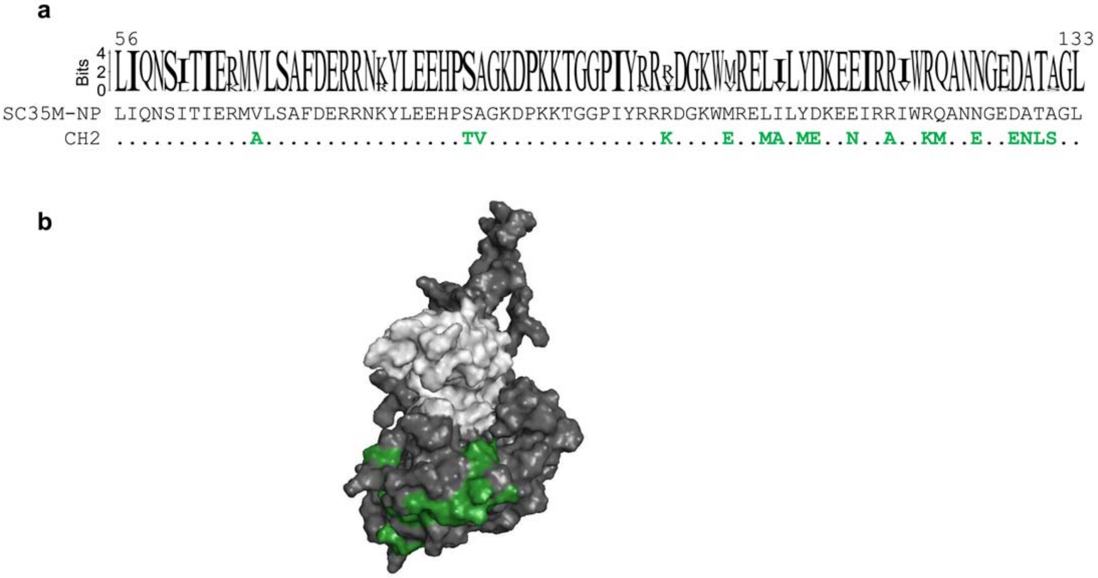

Supplementary Figure 2. Location and conservation of the bat influenza A-like NP amino acid in CH2

(a) Alignment (amino acid 56 to 133) of SC35M NP and CH2 harboring 18 Bat NP-specific amino acids. Upper panel shows the sequence logo of the consensus sequence of NP of available IAV strains (n = 27,675 strains).
(b) Positions of Bat-NP-specific amino acids in the modeled crystal structure of SC35M NP. The program PyMOL was used to assign the indicated positions. The body and head domains of NP are colored in dark and light grey, respectively. Bat specific amino acids are located in the body domain of NP and marked in green.

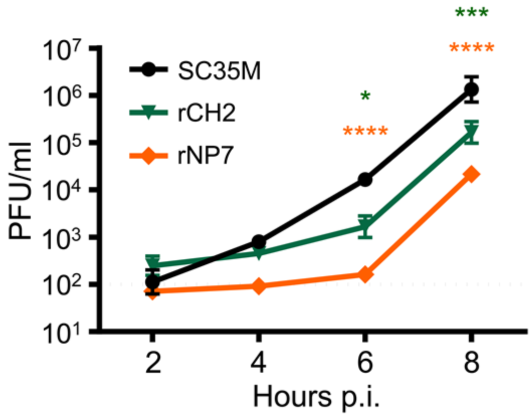

Supplementary Figure 3. Single cycle growth behavior of rCH2, rNP7 and wt SC35M.

MDCKII cells were infected at an MOI of 5 with the indicated viruses and time points post infection (p.i.). Virus titers were determined by plaque assay. Student's t test was used for two-group comparisons. Error bars indicate the mean and SD of 3 independent experiments. *P < 0.05; ***P < 0.001, ****P < 0.0001.

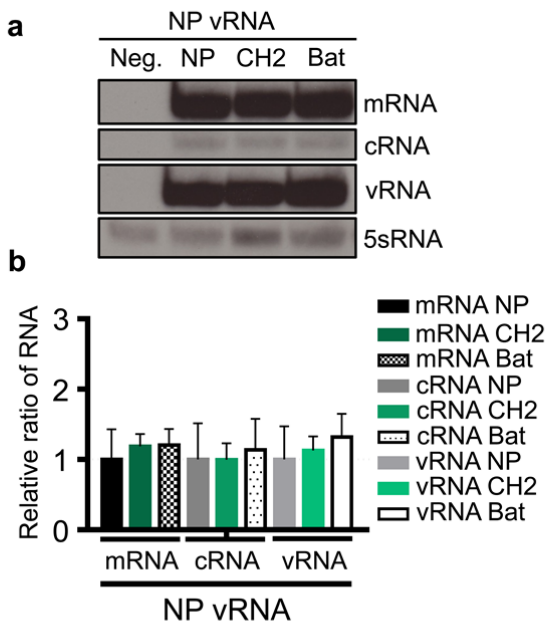

Supplementary Figure 4. SC35M polymerase reconstituted with CH2 efficiently replicates the NP segment.

(a) HEK293T cells were transiently transfected with expression plasmids coding for PB2, PB1, PA of SC35M, the indicated NP proteins, the SC35M NP segment with a mutation in the initiation codon (ATG to ACG) to prevent synthesis of segment-encoded NP. In the negative control (Neg.) PB1 was omitted. Steady state levels of viral transcripts (mRNA, cRNA and vRNA) and 5S ribosomal RNA (5S rRNA) were determined by primer extension analysis using total RNA 24 hours post transfection.
(b) Signal intensities obtained in panel (a) were normalized to the signal intensities obtained with 5S rRNA. Normalized values obtained with wt SC35M NP were set to 1 (all non-significant). Student's t test was used for two-group comparisons. Error bars indicate the mean and SD of 3 independent experiments.

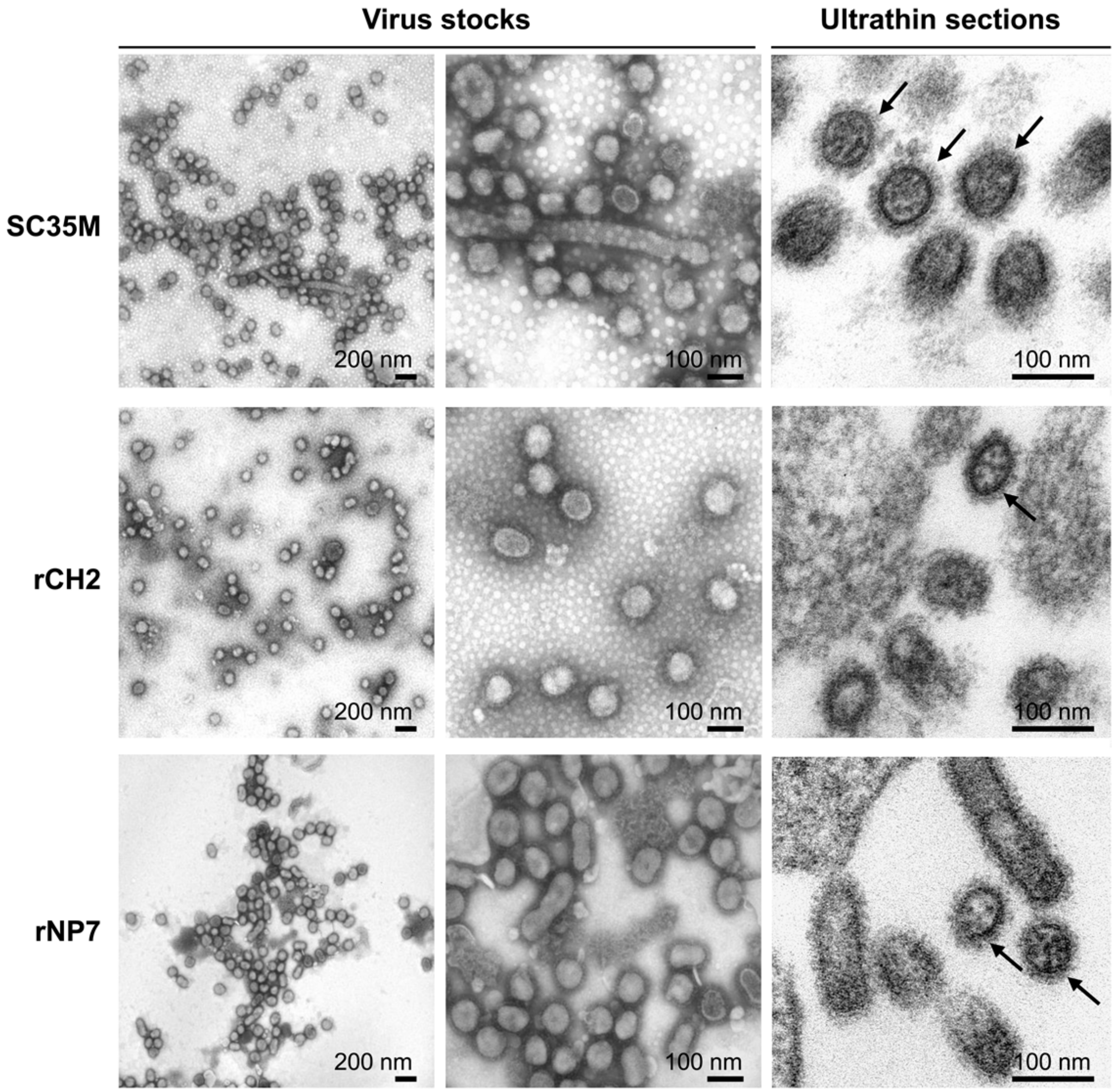

Supplementary Figure 5. Analysis of viral particles by transmission electron microscopy (TEM).

Depicted are representative micrographs of concentrated virus stocks and ultrathin sections of infected MDCK II cells. Virus particles with transversely sectioned vRNA segments are indicated by arrows.

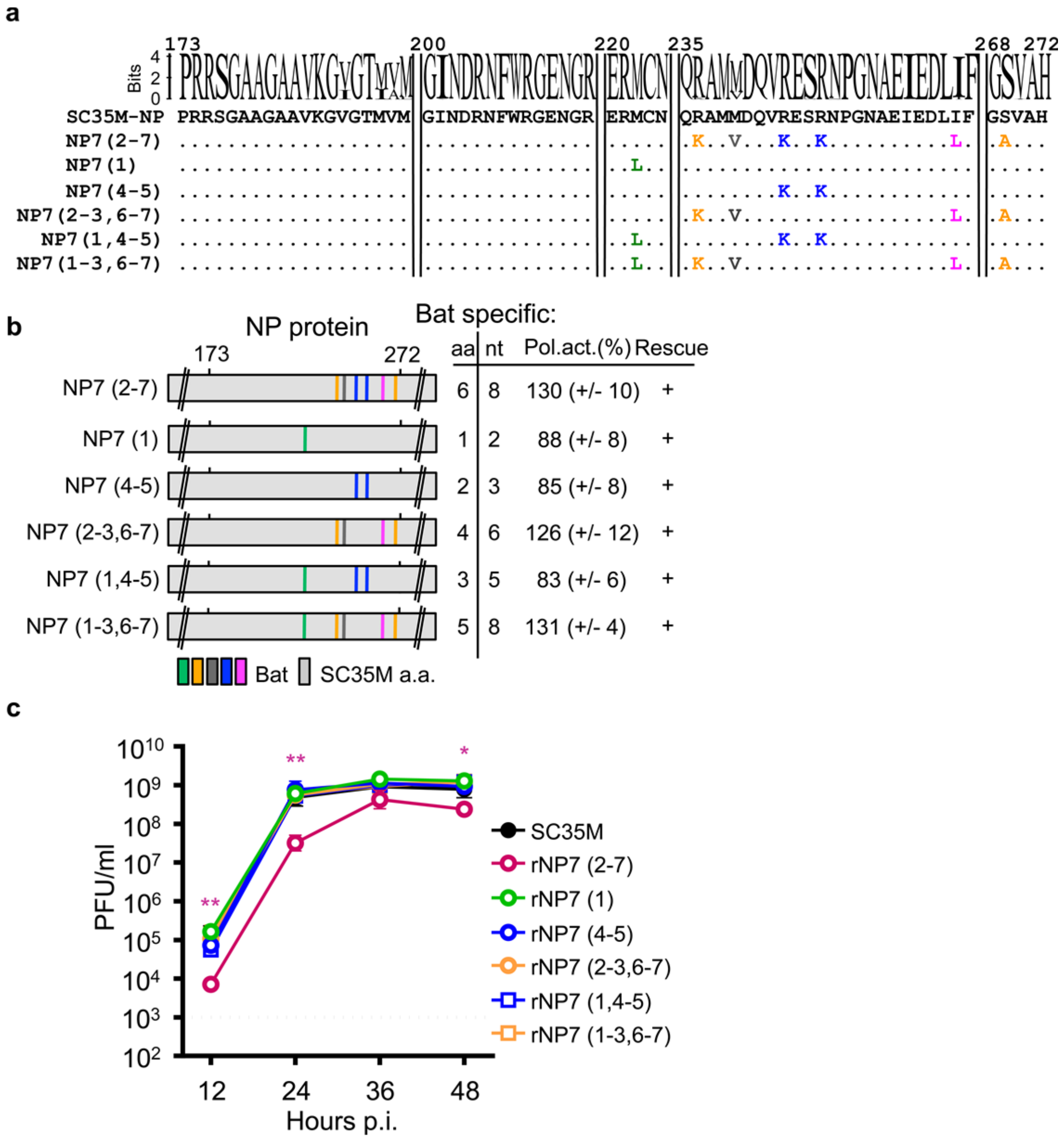

Supplementary Figure 6. Identification of Bat NP-specific amino acids in NP7 that cause attenuation.

(a) Alignment of SC35M NP and NP7 mutant proteins. The bat NP-specific amino acids are highlighted in the color code used in Fig. 3c. Upper panel shows the sequence logo of the consensus sequence of NP of available IAV strains (n = 27,675 strains).

(b) Ability of the indicated NP mutant proteins to support polymerase activity and rescue of SC35M. Relative SC35M polymerase activities in the presence of the mutant NP proteins were determined by polymerase reconstitution assays in HEK293T cells and values represent the relative polymerase activity. Numbers in parenthesis indicate the mean and SD of at least 3 independent experiments. Bat specific amino acids and nucleotides are indicated. SC35M rescue experiments were performed with NP segments encoding the indicated mutant proteins. + successful rescue.

(c) MDCKII cells were infected at an MOI of 0.001 with wt SC35M or mutant viruses. At the indicated time post infection (p.i.), virus titers were determined by plaque assay. Student's t test was used for two-group comparisons. Error bars indicate the mean and SD of at least 3 independent experiments. $^{*}P < 0.05$; $^{**}P < 0.01$.

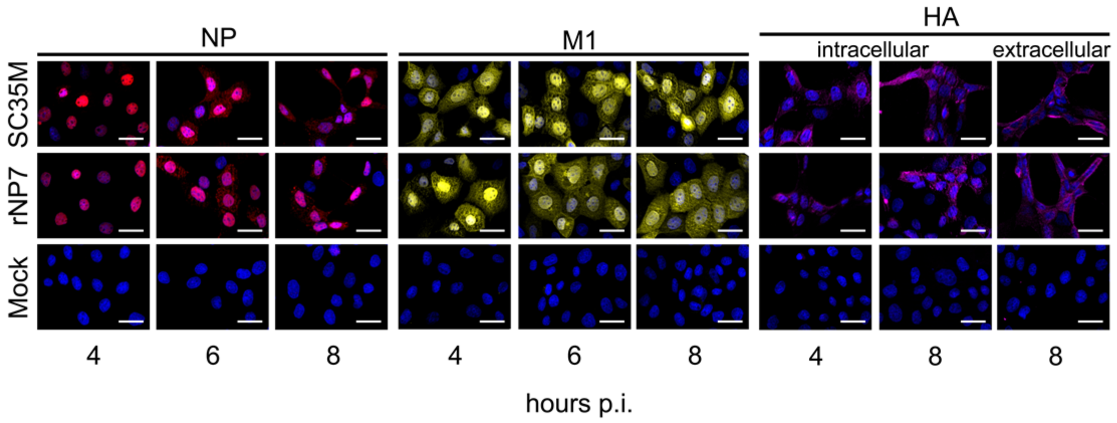

Supplementary Figure 7. Effects of Bat NP-specific amino acids encoded by rNP7 on subcellular localization of viral proteins.

Localization of NP, M1 or HA in MDCKII cells infected with the indicated viruses at an MOI of 5 for 4, 6 and 8 hours. For surface staining of HA cell were only fixed and not permeabilized. Scale bar: 10µM.

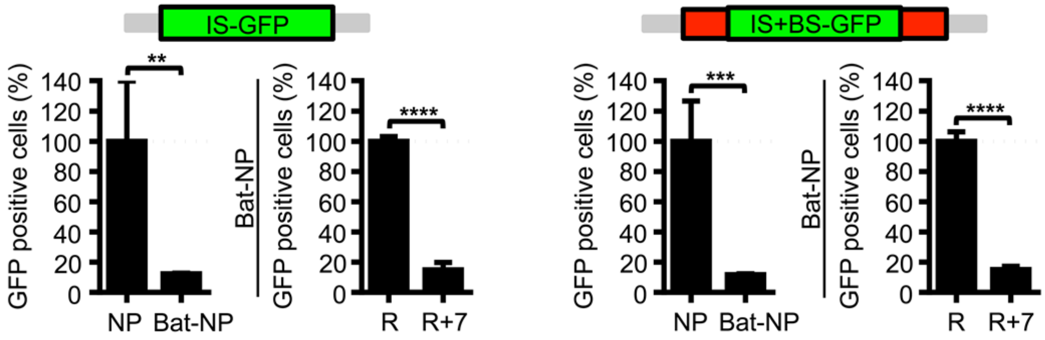

Supplementary Figure 8. Bat NP causes impaired genome packaging of SC35M

Packaging efficiency mediated by SC35M NP, or Bat NP in the presence of the reporter segment IS+GFP (left panel) or IST+BS-GFP (right panel) and if indicated in the presence of the remaining 7 wild type genome segments (+7). **P < 0.01, ***P < 0.001, ****P < 0.0001. R, reporter genome. Error bars indicate the mean and SD of at least 3 independent experiments

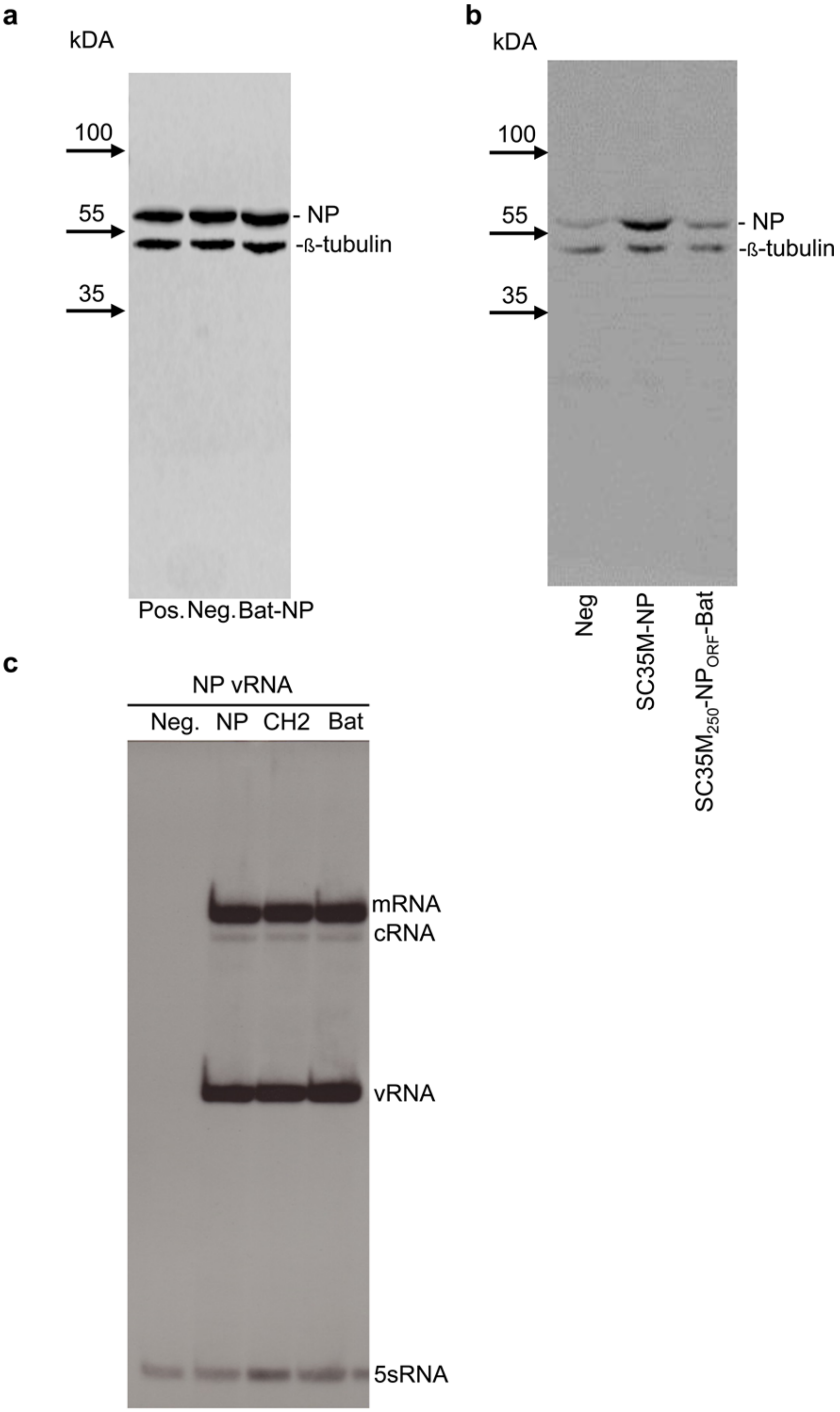

Supplementary Figure 9. Uncropped scans of Western blot and primer extensions

Supplementary Table 1. NP chimeras.

Name	Primers used for cloning*	GenBank accession numbers
CH1	01;02;03;04;05;06	KU860467
CH2	01;02;03;04;07;08	KU860468
CH3	01;02;05;06;07;08	KU860469
CH4	09;10;11;12;13;14	KU860470
CH5	01;02;05;06	KX344897
CH4.14	15;16;17;18	KU860471
CH4.4	18;19;20;21	KU860472
CH4.0	22;23	KU860473
NP14	18;24;25;26	KU860474
NP10	24;27;28;29	KU860475
NP7	18;24;30;31	KU860476
NP7(2-7)	18;24;32;33	KU860477
NP7(4-5)	18;24;34;35	KU860479
NP7(2-3,6-7)	18;24;34;36	KU860480
NP7(1,4-5)	18;24;35;37	KU860481
NP7(1-3,6-7)	18;24;36;37	KU860482
NP7-R31G	18;24;38;39	KU860483

* Numbered accordingly to Supplementary table 2.

Supplementary Table 2. DNA oligos used for cloning.

Nr.	Name	Sequence (5´→3´)
01	821-F	GATCGCGGCCGCACCATGGCGTCTCAAG
02	810-R	GATCCTCGAGTTAATTFTCATATTCCTCTGCATTG
03	56-F	CTGATCCAAAACAGCATAACAATTGAGAGGATGG
04	56-R	CCATCCTCTCAATTGTTATGCTGTTTTGGATCAG
05	289-F	GACTTTGAAAGAGAGGGATACTCTCTAGTCGG
06	289-R	CCGACTAGAGAGTATCCCTCTCTTTCAAAGTC
07	130-F	CTGGTCTCACCCATTTGATGATCTGGCATCCAACTGAAT
08	130-R	ATTCAAGTTGGAGTGCCAGATCATCAAATGGGTGAGACCAG
09	5-Fr1	CCATGGCGTCTCAGGGAGCGAAAGCAGGGTAGATAATCACTCACCGAGTG
10	3-Fr1	CCATGGCGTCTCAAGCATCATTCAGGTTGGAATGCCAGAT
11	5-Fr2	CCATGGCGTCTCATGCTACCTATCAGCGAACAAGAGCCC
12	3-Fr2	ACAAGGCGTCTCAGGCATGACTTATGAGCTACTGCTCCTCGGAG
13	5-Fr3	CCATGGCGTCTCATGCCTCCCAGCCTGTGTATATGGAC
14	3-Fr3	CCATGGCGTCTCATATTAGTAGAAACAAGGGTATTTTTCTTTAATTG
15	5-Fr1	GTTGATCCGGATGATCAAAAGAGGAATGAATGACAGAAATTTCTGGAAGGGTGAACAAGGGAGAAGAACTCGAATAGCTTATGAAAGGCTCTGTAACATCCTGAAAGGAAAGTTTCAAACAGCCGCACAG
16	3-Fr1	AGGGCAGATCTTGCAAGAAATAGTAAGTCCTCTATTTCTGCATTTCCTGGGTTTTTGCTTTCTTTGACTTGATCAACCATTGCTTTCTGTGCGGCTGTTTGAAACTTTCCTTTCAGGATGTTACAGAGCC
17	5-Fr2	CTTGCAAGATCTGCCCTGATCC
18	3-Fr2	ATCCACTGGCCACAGAAAGTCCATA
19	5-Fr1	GTTGATCCGGATGATCAAAAGAGGAATCAATGACAGAAATTTCTGGAGGGGTGAAAATGGGAGAAGAACTCGAATAGCTTATGAAAGGATGTGTAACATCCTGAAAGGAAAGTTTCAAACAGCCGCACAG
20	3-Fr1	AGGGCAGATCTTGCAAGAAATATTAAGTCCTCTATTTCTGCATTTCCTGGGTTTCTGCTTTCTCTGACTTGATCCATCATTGCTCGCTGTGCGGCTGTTTGAAACTTTCCTTTCAGGATGTTACACATCC
21	5-Fr2	ATTTCTTGCAAGATCTGCCCTGATCCTCCGAGGATCAGTAGCTCATAA
22	5-Fr1	TAATCACTCACCGAGTGACATCCACATCATGGCGTCCCAAGGCACC
23	3-Fr1	GATCATCCGGATCAACTCCATTACCATGGTCCCCACTCCTTTGACTGCTGCCCCAGCTGCTCCAGATCTTCTTGG
24	5-Fr1	ACCATGGCGTCTCAAGGCACCAAACGATCT
25	3-Fr1	CCTCGCTTGATCATCCGAATCAAT
26	5-Fr2	GATCAAGCGAGGGATGAATGATCGGAATTTCTGGAAAGGCGAACAAGGGCGGAGAACAAGAATTGCATACGAGAGACTGTGTAACATTCTCAAGGGGAAATTTCAAACAG
27	3-Fr2	CAGCACAAAAAGCAATGGTGGACCAGGTGAAGGAAAGCAAGAATCCTGGGAATGCTGAAATTGAAGATCTCCTCTTTCTTGCACGGTCTGCTCTCATTCTGAGGGGAGCAGTGGCTCATAAGTCCTGCCTGCCTGCTTGTGTGTATGGACTTTCTGTGG
28	3-Fr1	AATTTGCGTCTCTCCATTATGAGTGTTCCAACCCCTTTCACTGCAGTTCCAGCAGCCCCAGCCCTCCTTGG
29	5-Fr2	ATTATGGCGTCTCAATGGAATTGATTCGGATGATCAAGCGAGGG
30	3-Fr1	ACCATGGCGTCTCAGATCATCCGAATCAATTCCATTACCATTGTTC
31	5-Fr2	AACCGGCGTCTCAGATCAAGCGAGGGATCAATGATCGGAATTTCTGGAGAGGCGAAAATGG
32	3-Fr1	CCATGGCGTCTCACTGTTTGAAATTTCCCCTTGAGAATGTTACACATTCTCTCGTATGCAAT
33	5-Fr2	CCATGGCGTCTCAACAGCAGCACAAAAAGCAATGGTGG
34	3-Fr1	ACCATGGCGTCTCACTGTTTGAAATTTCCCCTTGAG

35	5-Fr2	CCATGGCGTCTCAACAGCAGCACAACGAGCAATGATGGACCAGGTG AAGGAAAGCAAGAATCC
36	5-Fr2	CCATGGCGTCTCAACAGCAGCACAAAAAGCAATGGTGGACCAGGTGA GGGAAAGCCGGAATCCTGGG
37	3-Fr1	CCATGGCGTCTCACTGTTTGAAATTTCCCCTTGAGAATGTTACACAGT CTCTCGTATGCAAT
38	3-Fr1	AACCGGCGTCTCTAACCATTCCCCCAACAGATGCTCTG
39	5-Fr2	ACCATGGCGTCTCAGGTTGGTGGAATCGGGAGATTCT

Supplementary Table 3. Nucleotide composition of NP chimera CH1, CH2, CH3, CH4 and CH5 based on the positive sense of the respective genome segments.

Name	5´ end of SC35M NP (nt)	Bat NP ORF (nt)	3´ end of SC35M NP (nt)
CH1	1-215	230-914	937-1565
CH2	1-215	230-435	437-1565
CH3	1-464	467-919	937-1565
CH4	1-485	476-859	886-1565
CH5	1-903	903-1565	

Supplementary Table 4. DNA oligos used for primer extension analysis.

Primer		Sequence (5’-3’)	Binding site*	Size (nt)
PB2	vRNA	TGCTAATTGGGCAAGGAGAC	2198-2217	143
	cmRNA	GCCATCATCCATTTCATCCT	163-182	182-195
PB1	vRNA	TGACTTCGAGTCTGGACGGA	2208-2227	133
	cmRNA	TCCATGGTGTATCCTGTTCC	127-146	146-159
PA	vRNA	GCCTGATTAATGATCCCTGGG	2102-2122	131
	cmRNA	CACCCCGTTCATCAACGAAATGG	177-199	199-212
HA	vRNA	GGTTTAGCTTCGGGGCATC	1610-1628	130
	cmRNA	CCCGATATTCGCGGTTTCCAC	172-192	192-205
NP	vRNA	GATGTGTCTTTCCAGGGGCG	1408-1427	157
	cmRNA	GCCTCCCTTCATAGTCGCTG	192-211	211-224
NA	vRNA	TGGACGAGCAACAGCTTAGT	1340-1359	121
	cmRNA	CGTCGTGTTGTTCTGTGTTATGC	175-196	196-209
M	vRNA	TACGGATTGAAAAGAGGGC	882-900	147
	cmRNA	TTCCCTTAGTCAGAGGTGAC	181-200	200-213
NS	vRNA	GTTTGAAGAAATAAGGTGGC	714-733	176
	cmRNA	GCTGTTTCGATGTCCAGACC	177-196	196-209

* based on 5’ to 3’ numbering of the template sequence in the positive sense.

Supplementary Table 5. SC35M segment-specific primers for quantitative RT-PCR.

Segment	Direction	Sequence (5’-3’)
PB2	F	GCACAGGATGTAATCATGGAAGT
	R	ACCAATTCTCTTTCTAGCATGTATGC
PB1	F	CCAGACCTATGATTGGACATTAAACA
	R	CCGTTCGATCTGAAGACCTCTATAGT
PA	F	AATGATCCCTGGGTTTTGCTTA
	R	TGCCACAGCTATTTCAGTGCAT
HA	F	GCTTTTGGGAGCAATTGCTGG
	R	GTGCTTTTGTAGTCAGCTGCA
NP	F	CTGATCCAAAACAGCATAACAATTGAGAGGATGG
	R	ATTCAAGTTGGAGTGCCAGATCATCAAATGGGTGAGACCAG
NA	F	CGTTCCAGAAGTGGTTTTGAGATG
	R	AAACTCCCTGAGTATCCTGACC
M	F	CTTCTACCGAGGTCGAAACG
	R	AGGGCATTTTGGACAAAKCGTCTA
NS	F	GGACCAGGCGATCATGGATAA
	R	CAATTGCTCCTTCTTCGGTGAA
